# Draft Genome Sequences of Closely Related Listeria monocytogenes Lineage III Strains Isolated from a Food Processing Environment and a Case of Human Listeriosis

**DOI:** 10.1128/mra.00250-23

**Published:** 2023-05-22

**Authors:** Phillip Brown, Rebecca Kanenaka, Yi Chen, Mirena Ivanova, Pimlapas Leekitcharoenphon, Driss Elhanafi, Sophia Kathariou

**Affiliations:** a Department of Plant and Microbial Biology, North Carolina State University, Raleigh, North Carolina, USA; b Department of Health, Laboratories Division, Honolulu, Hawaii, USA; c Division of Microbiology, Center for Food Safety and Applied Nutrition, Food and Drug Administration, College Park, Maryland, USA; d Research Group for Genomic Epidemiology, National Food Institute, Technical University of Denmark, Lyngby, Denmark; e Biomanufacturing Training and Education Center, North Carolina State University, Raleigh, North Carolina, USA; f Department of Food, Bioprocessing and Nutrition Sciences, North Carolina State University, Raleigh, North Carolina, USA; University of Maryland School of Medicine

## Abstract

Listeria monocytogenes lineage III is genetically highly diverse, and closely related lineage III strains from food facilities and human listeriosis have not been reported. Here, we report the genome sequences of three closely related lineage III strains from Hawaii, namely, one isolated from a human case and two isolated from a produce storage facility.

## ANNOUNCEMENT

Most human listeriosis infections involve food contaminated with Listeria monocytogenes lineage I and II, with serotype 4b making major contributions (1). Serotype 4b lineage III strains are occasionally implicated in human listeriosis (2, 3), but descriptions of closely related lineage III strains isolated from clinical and food-related sources are lacking. Strains A2 and B2 were isolated in 1999 from a walk-in refrigerator in a produce storage facility in Hawaii, while strain (HI) 267 was isolated from a human listeriosis case also in Hawaii in 1999 (Table 1). All three strains were lineage III, serotype 4b, sequence type 1264 (ST 1264), and clonal complex 1264 (CC 1264), with the same internal deletion in *inlA*, and the same type I-B CRISPR subtype, with 26 spacers of identical sequence and order (Table 1). A2 and B2 were identical via core genome multilocus sequence typing (cgMLST) and differ from (HI) 267 by 31 cgMLST alleles. The genomic DNA of all three strains was resistant to restriction by Sau3AI but susceptible to MboI digestion. Of special interest was a novel type II Sau3AI-like restriction modification (RM) system with specificity for methylated cytosines at GATC sites. This RM system was rare among other L. monocytogenes genomes in the NCBI database and in the same genomic region but distinct (45.6% nucleotide identity) from its counterpart in CC1 and other L. monocytogenes (4). It was approximately 1.5 kb apart from another type II RM system, LmoJ2, with specificity for GCWGC (W = A or T) (5).

**TABLE 1:** Listeria monocytogenes strains investigated in this study

Characteristic	Data by strain		
	(HI) 267	A2	B2
Source	Clinical, HI	Environmental, HI	Environmental, HI
Isolation yr	1999	1999	1999
Serotype	4b	4b	4b
Lineage	III	III	III
Sequence type	1264	1264	1264
Clonal complex	1264	1264	1264
Restriction modification system cluster	Type II LmoJ2 and Type II Sau3AI-like	Type II LmoJ2 and Type II Sau3AI-like	Type II LmoJ2 and Type II Sau3AI-like
CRISPR subtype	Type I-B	Type I-B	Type I-B
No. of CRISPR spacers	26	26	26
Internalin A	Internal deletion (aa 737–739)	Internal deletion (aa 737–739)	Internal deletion (aa 737–739)
No. of contigs	17	29	35
Total length (bp)	2,872,927	2,886,226	2,907,196
*N*_50_ (bp)	526,919	240,424	160,021
Coverage (×)	88	92	89
No. of reads	1,010,778	900,845	877,979
GC content (%)	38.0	38.0	38.0
Sequencing method	Illumina MiSeq	Illumina NextSeq 500	Illumina NextSeq 500
Read quality control and trimming tool	CLC Genomics Workbench 7.5.1	FastQC v0.11.5, bbduk2 v38.89	FastQC v0.11.5, bbduk2 v38.89
GenBank accession	ABJMII000000000	DAKXYS000000000	DAKXVF000000000
BioSample accession	SAMN31821600	SAMN31927805	SAMN31927806
Assembly accession	GCA_026274475.1	GCA_026848395.1	GCA_026846215.1
Sequence Read Archive accession	SRR22362541	SRR22444782	SRR22444781

Strains A2 and B2 were isolated from enrichments of environmental swabs of a produce storage facility following incubation of enrichments on Oxford agar and lithium chloride-phenylethanol-moxalactam (LPM) agar at 35°C for 24 to 48 h as described (6). Strain (HI) 267 was provided by the Hawaii Department of Health. Strains were stored as frozen stocks at −80°C, streaked onto brain heart infusion (BHI) agar (Becton, Dickinson & Co.), and incubated at 37°C overnight for single-colony isolation. Genomic DNA was extracted from isolated single colonies grown overnight at 37°C in BHI broth by the DNeasy blood and tissue kit (Qiagen, Valencia, CA). Libraries were prepared using 0.5 to 1 ng of genomic DNA with a Nextera XT DNA library preparation kit (Illumina, San Diego, CA). Genomes were sequenced using either a NextSeq 500 sequencer with the NextSeq 500/550 high-output kit v2.5 (300 cycles, 2 × 150 bp) (Illumina) or a MiSeq desktop sequencer with the MiSeq kit v2 (500 cycles, 2 × 250 bp) (Illumina) according to the manufacturer’s instructions (Table 1). Raw sequencing reads were quality controlled by FastQC v0.11.5 (7) and trimmed using bbduk2 from bbtools v38.89 (http://sourceforge.net/projects/bbmap/) or using CLC Genomics Workbench 7.5.1 (CLC Bio, Boston, MA) (Table 1). Reads were deposited into the National Center for Biotechnology Information (https://www.ncbi.nlm.nih.gov), assembled by SKESA v2.2 (8), and assemblies were annotated using the NCBI Prokaryotic Genome Annotation Pipeline (PGAP) v6.3 (9). ST and CC designations were assigned using BIGSdb-Lm, hosted by Institut Pasteur (https://bigsdb.pasteur.fr/listeria/) (10). Default parameters were used for all software. DNA restriction digests were performed as described previously (11).

### Data availability.

This whole-genome shotgun project for (HI) 267, A2, and B2 has been deposited in GenBank under the accession numbers ABJMII000000000.1, DAKXYS000000000.1, and DAKXVF000000000.1, respectively (Table 1). The versions described in this paper are the first versions.
